# What to Do with Our Sons

**Published:** 1884-03

**Authors:** 


### What to Do with Our Sons.

A very wise M. R. C. S. thus writes to the *Med. Times and Gaz.:*

"I am a professional man with a good income, but I have a large family of daughters and two sons. Knowing how precarious are the chances of success in any of the learned professions, I have just apprenticed my youngest son, aged sixteen years, to a builder. Of course, he has to work at the bench, and go out with the workmen 'on jobs,' but he is happy, and his time well employed. When he is twenty-one, he will have become master of his trade, and, being a well educated lad, and sharp to boot, a very few pounds would start him in one of the colonies on the high road to competency. This is what I do with 'our boys'—that is, for those who are handy with their tool-chest, and English lads are. The silly pride of parents is the chief drawback to their sons' success in life."

